# The Effect of Physical Exercise on Depression in College Students: The Chain Mediating Role of Self-Concept and Social Support

**DOI:** 10.3389/fpsyg.2022.841160

**Published:** 2022-05-16

**Authors:** Junliang Zhang, Shuang Zheng, Zhongzheng Hu

**Affiliations:** College of Science and Technology, Nanchang Hangkong University, Jiujiang, China

**Keywords:** physical exercise, depression, self-concept, social support, college students

## Abstract

**Objective:**

This study introduced self-concept and social support as research variables to establish a research mechanism, in order to encourage college students to participate in sports better, relieve or overcome depression.

**Methods:**

The survey was conducted among 1,200 college students in Jiangxi, China. Serial mediation models were used to examine whether self-concept and social support mediated in the effect of physical exercise on depression.

**Results:**

Physical exercise significantly negatively predicted college depression. Moreover, Self-concept and social support mediate the relationship between physical exercise and depression in college students.

**Conclusion:**

This study reveals how physical exercise affects college students’ depression and its mechanism, and the results have certain enlightenment significance for maintaining and promoting college students’ physical and mental health.

## Introduction

College students are the backbone of social and economic development in the future, and their growth and mental health have been the focus of public attention. With the quickening pace of society, pressure from all aspects increases, if there is no good anti-pressure, they will lead to depression and other psychological problems. Depression is a mental state with reduced energy, low mood, loss of interest, and poor quality of life. In severe cases, suicidal ideation and even suicidal behavior may occur (Ibrahim et al., 2013). The depression discussed in this study is not the diagnostic depression of mental disease, and only refers to a sub-health state. Studies at home and abroad have found that the number of suicides due to depression has been increasing year after year in recent years (Walker et al., 2015). If the administrators in the university department fail to recognize this phenomenon and take appropriate intervention measures, intervention measures in time, the development of the physical and mental health of college students will be seriously hindered.

Physical exercise refers to any physical activity that promotes physical and mental development by means of physical movement (Morgan and Ellickson, 1989). People who engage in regular physical exercise have a high level of self-confidence and values, leading to greater satisfaction with life, and thus reduced anxiety and depression (Esmaeilzadeh, 2015; Wu et al., 2016). Rebecca et al. ‘s study on 467 adolescents showed that although physical activity had an intervention effect on adolescents’ sleep quality and depression, it had no predictive effect on depression level (Slykerman et al., 2019). Salmon’s study found that aerobic exercise has anti-depression and anti-anxiety effects, but strenuous physical exercise has a confusing effect on emotions, which has both positive and negative effects (Salmon, 2001). Strong evidence shows that habitual physical activity has not been proven to prevent depression, but increasing moderate physical activity can significantly reduce symptoms of depression and anxiety, and improve self-esteem and a positive outlook on life (Crone, 2003; Sun et al., 2014). Khanzada conducted a descriptive statistical analysis of 269 individuals aged 18-45. In the studied adult population, women are more prone to depression than men, and those who exercised regularly had lower rates of depression than those who did not and physical exercise is significantly correlated with a lower frequency of anxiety and depression (Khanzada et al., 2015). In conclusion, we proposed a hypothesis that Physical exercise had a positively predictive effect on depression of college students (H1).

To better investigate the effect of physical exercise on the depression of college students, self-concept was introduced as a mediator based on a literature review. Self-concept refers to a person’s view of himself or herself, which can also be defined as the sum of everyone’s perception of himself or herself, and plays a guiding role in in everyone’s mental function (Simons et al., 2012). Abundant research results have proven the positive correlation between participation in programs of physical activity and higher levels of physical self-esteem and global self-concept (Perikles, 2006). Several studies show that the influence of self-concept, physical exercise satisfaction, quality of life, and self-concept are related, that is, the university sports exercise satisfaction is higher, the higher the quality of life and self-concept. According to a cognitive theory of depression, if individuals have low self-concept clarity, their cognitive abilities are reduced and they develop or maintain depression (DING Qian, 2016). At the same time, empirical studies have shown that college students’ self-concept has a negative predictive effect on depression (Fan and Yuan, 2001; Li, 2004). Thus, we hypothesized that self-concept is the mediating variable between physical exercise and depression in college students (H2).

How does college students’ self-concept affect their depression? By reviewing relevant literature, this study intends to use social support as a mediator between depression and self-concept in college students. Social support refers to beneficial interpersonal relationships from friends, family members, other contacts, or with a larger society. Social support is more about generating a feeling that a person is cared for and loved, respected and valued, or is part of a network of mutual interpersonal commitment. Empirical studies show that individuals with low self-concept clarity are difficult to change peer support, and social support and self-concept are significantly positively correlated (Emery et al., 2015; Quinones and Kakabadse, 2015). According to the self-categorization theory of depression cognition, depression can only be affected when the corresponding depression cognition interacts with a specific interpersonal relationship, that is, the interaction between negative cognition and negative interpersonal relationship can predict depression (Abramson, 1989). A good amount of empirical studies have also found that self-concept not only has a direct effect on depression (Wang and Qian, 2010), but also indicates the existence of other mediating variables, among which social support is one of the influencing factors. Therefore, we hypothesized social support might mediate the relationship between self-concept and depression (H3).

According to the interaction theory of social symbols, society is composed of interacting individuals who construct corresponding social roles, relationships, and standards. Physical exercise is a social interaction involving not only individuals but also groups (Mead, 1934). Some studies have pointed out that the interaction between individuals, family, and friends can promote the acquisition of the instrumental support, help maintain the level of physical exercise and active participation, so as to change the lifestyle and improve the level of mental health (Semmer et al., 2008). A large number of studies have shown that exercise can relieve depression and improve adverse health effects; at the same time, the study found that in the process of physical exercise, combined with their own interest and hobby to interact with different people, virtually promotes its own social ability, friendship, support, understanding social support, so as to enhance the self-worth and self-esteem, which reduces negative emotions, such as depression (Tang et al., 2007; Xu, 2011). Furthermore, Maslow’s hierarchy of needs theory believes that friends, family, and other communication are the basic needs of belonging and communication. When an individual’s needs are not being met, he or she will have negative emotions, such as anxiety and depression. Therefore, when the needs for interpersonal communication are not met, individuals will get less social support, which will easily lead to depression (Maslow, 1964). Therefore, this study hypothesized that social support is a mediating variable between physical exercise and depression in college students (H4).

**FIGURE 1:**
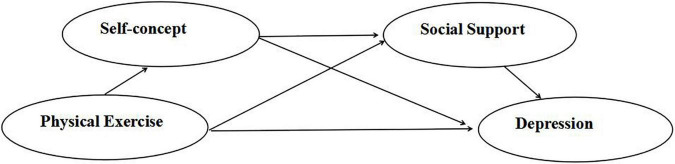
The chain-mediated modle of self-cocept and social support.

In conclusion, this study intends to explore the influence mechanism of physical exercise on college students’ depression, and construct a chain mediation model of physical exercise on college students’ depression with social support and self-concept as mediating variables. The hypothesis model is shown in Figure 1.

## Materials and Methods

### Study Design and Participants

Participants were recruited from undergraduates at some universities located in Jiangxi province, China. Due to the impact of COVID-19, the researchers asked the participants to answer an online questionnaire. Among the collected questionnaires, 1097 of which were valid, with an effective rate of 90%, with 440 male students accounting for 41 percent and 639 female students accounting for 59 percent. The subjects ranged in age from 17 to 24 (21.53 ± 0.84).

### Measurements

#### Depression Scale

This study adopted the Self-Rating Depression Scale (the version by Zhang (1997)). This consists of 20 items using the 4-point Likert scale ranging from “no or little time”, to “most or all time”. The lowest score is 0, and the highest score is 60. The higher the score is, the more serious the degree of depression. In the actual measurement, the internal consistency coefficient of the questionnaire is 0.83.

#### Physical Activity Scale

The Physical Activity Scale was used to measure the level of the participants’ physical activity. It was based on the Physical Exercise Intensity Scale for College Students (Liang, 1994). The scale was divided into three dimensions with 14 items: Intensity, Duration, and Frequency of Exercise. Higher scores indicate more intense exercise. In the current study, the scale’s Cronbach’s alpha was 0.86.

#### Social Support Scale

The Social Support Comprehension Scale adapted by Jiang Ganjin was adopted in this study, which consists of 12 items. The scale contains three dimensions:Family Support, Friend Support and Others. Respondents agreed to use a seven-point Likert scale that ranged from “very dissatisfied” to “dissatisfied.” In the study, the final scale’s Cronbach’s alpha was 0.87.

#### Self-Concept Scale

Self-concept was evaluated by the self-concept scale, which was developed by Zhou based on the Self-concept clarity scale (Niu et al., 2016). The questionnaire was scored on a 7-point Likert scale ranging from 1,” very dissatisfied “to 7,” dissatisfied”. Cronbach’s coefficient of this measure in this study was 0.90.

#### Data Analysis

SPSS24.0 and macro program PROCESS were used to sort out and analyze the data for depression, physical activity, social support and self-concept. SPSS was first used for correlation analysis, and then PROCES model 6 was used to analyze the mediation effect.

## Results

### Common Method Biases

Since the data in this study were filled in by the anonymous self-report method of the subjects, there may be a common method bias, so the Harman single-factor test method was used to test the data for bias. The factors with eigenvalues greater than 1 were extracted by testing, and it was found that there were 11 factors greater than 1, and the variation explained by the first factor was 22.88%, which was less than 40% of the critical value standard, indicating that there was no serious common method deviation in the data in this study.

### Descriptive Statistics and Correlation Analyses

Table 1 reports descriptive statistics and correlative statistics among variables. Physical exercise was negatively correlated with depression (*r* = −0.221, *p* < 0.01), and was positively related to self-concept (*r* = 0.291, *p* < 0.05), social support (*r* = 0.1611, *p* < 0.01).

**TABLE 1:** Pearson correlation analysis among different variables.

	M ± SD	1	2	3	4
physical exercise	8.04 ± 2.47	1			
depression	37.14 ± 7.77	−0.221**	1		
social support	62.37 ± 13.01	0.161**	−0.529**	1	
self-concept	48.92 ± 9.56	0.291**	−0.326**	0.143**	1

*p < 0.05, **p < 0.01, ***p < 0.001.

### Mediation Analyses

In order to better explore the mechanism of physical exercise on depression of college students, self-concept and social support were considered to be the mediators. We use Model 6 of PROCESS to test the mediating effect.

Based on the structural equation, the chain mediating effect of self-concept and social support was further tested by Bootstrap (repeated sampling 5,000 times) at the deviation corrected percentile. As presented in Figure 1 and Table 2, physical exercise was a direct positive predictor of self-concept (β = −0.38, *P* < 0.001), self-concept negatively predicted depression (β = −0.27, *p* < 0.001), and the mediating effect of self-concept on physical exercise on depression was 0.103, so H1 was supported. Physical exercise significantly positively predicted social support (β = −0.13, *P* < 0.01), social support negatively predicted depression (β = −0.54, *P* < 0.001), and the mediating effect of social support on the effect of physical exercise on depression was 0.07, thus H2 was established. At the same time, self-concept positively predicted social support (β = −0.11, *P* < 0.01), and the mediating effect of self-concept and social support on the effect of physical exercise on depression was 0.023, and the results also showed that physical exercise had a negative predictive effect on the depression of college students (β = −0.08, *P* < 0.05), the direct effect was 0.08, so H3 was valid, which indicates the mediating role of self-concept and social support. It can be seen that self-concept and social support play a part of mediating chain role in the effect of physical exercise on depression of college students.

**TABLE 2:** Bootstrap mediating effects of social support and self-concept.

Paths	Boot SE	Boot LLCI	Boot ULCI	Effect of SUM
PE - > DS	−0.08	−0.13	−0.02	
PE - > SC- > DS	−0.103	−0.18	−0.05	0.37
PE - > SS- > DS	−0.070	−0.10	−0.03	0.25
PE - > SC- > SS- > DS	−0.023	−0.03	−0.003	0.08
Total Effect	−0.196	−0.20	−0.11	

*PE, physical exercise; DS, depression; SC, self-concept; SS, social support.*

*P < 0.05,**P < 0.01 and ***P < 0.001.

## Discussion

A cross-sectional study was conducted to understand the relationship between physical exercise, depression, self-concept, and social support, and analyze the chain mediating role of self-concept and social support.

### The Influence of Physical Exercise on Depression of College Students

This study shows that physical exercise can negatively predict the depressed mood of college students, which is obviously consistent with the previous result that physical exercise is negatively correlated with the risk of depression (Edman et al., 2014). Therefore, we should improve the physical exercise level of college students to reduce depression and other negative emotions of college students (Ruby et al., 2011). Empirical studies have shown that by testing the time, intensity, and frequency of college students’ physical exercise, moderate to large amounts of physical exercise have a great effect on alleviating negative emotions, that is, college students with moderate to large amounts of physical exercise have a lower level of anxiety and depression [36]. Studies have also shown that both aerobic exercise and traditional physical activities have positive effects on the improvement of individual depression (Rimer et al., 2012). Therefore, we should attach great importance to the impact of physical exercise on college students’ depression.

### Mediating Effects of Self-Concept and Social Support

This study shows that self-concept and social support play a chain intermediary role in the relationship between physical exercise and depression, indicating that the mediating effect of self-concept and social support was established. In the model of the study, physical exercise can not only directly affect the college students’ levels of depression, but also indirectly, by self-concept and social support, affect college students’ depression.

It can be concluded that physical exercise could alleviate college students’ depression mood and their self-concept cognition by means of physical exercise, so as to obtain greater social support and reduce the level of depression. Martinez pointed out in his study that the level of individual self-concept can indeed improve social perception through physical exercise, to better integrate into a peer group, and thus obtain more social support and alleviate depression (Yong and Tian, 2019). Many previous studies have also found that through physical exercise one can obtain more social support, relieve stress levels, and reduce adverse emotions, such as anxiety and depression (Salmon, 2001). Hence, it is necessary to guide our students to engage in more physical exercise, and to actively improve their self-concept awareness and social support level, so as to reduce their depression.

### Implications

Physical exercise can positively predict college students’ depression and improve their positive emotions through appropriate physical activities. The mediating effect of self-concept and social support was also found in our study. Therefore, in addition to actively improving the level of self-cognition of college students, it is necessary to improve their interpersonal communication skills, so as to reduce the level of depression.

### Limitations and Future Studies

We can still find deficiencies in the study, which need to be improved in the future research. This study only discusses the influence of physical exercise on depression from the perspective of psychology, which can be discussed with the method of neuroscience in future research.

## Conclusion

This study shows that physical exercise has a negative effect on depression in college students’ depression, and social support and self-concept played a chain mediating role between physical exercise and depression. The mediating effect of self-concept and social support on depression indicates that the two variables have important practical significance in improving college students’ mental health. The study will provide a reference for educators to improve the healthy physique and psychological quality of college students in the future.

## Data Availability Statement

The raw data supporting the conclusions of this article will be made available by the authors, without undue reservation.

## Ethics Statement

Written informed consent was obtained from the individual(s) for the publication of any potentially identifiable images or data included in this article.

## Author Contributions

JZ and SZ conceived and designed the experiments and wrote the manuscript. JZ, SZ, and ZH carried out the protocol and the questionnaire survey and revised the manuscript. JZ analyzed the data. All authors have read and agreed to the published version of the manuscript.

## Conflict of Interest

The authors declare that the research was conducted in the absence of any commercial or financial relationships that could be construed as a potential conflict of interest.

## Publisher’s Note

All claims expressed in this article are solely those of the authors and do not necessarily represent those of their affiliated organizations, or those of the publisher, the editors and the reviewers. Any product that may be evaluated in this article, or claim that may be made by its manufacturer, is not guaranteed or endorsed by the publisher.
